# Negative Patient Descriptors: Documenting Racial Bias In The Electronic Health Record

**DOI:** 10.1377/hlthaff.2021.01423

**Published:** 2022-01-19

**Authors:** Michael Sun, Tomasz Oliwa, Monica E. Peek, Elizabeth L. Tung

**Affiliations:** University of Chicago, Chicago, Illinois.; University of Chicago.; University of Chicago.; University of Chicago.

## Abstract

Little is known about how racism and bias may be communicated in the medical record. This study used machine learning to analyze electronic health records (EHRs) from an urban academic medical center and to investigate whether providers’ use of negative patient descriptors varied by patient race or ethnicity. We analyzed a sample of 40,113 history and physical notes (January 2019–October 2020) from 18,459 adult patients for sentences containing a negative descriptor (for example, resistant or noncompliant) of the patient or the patient’s behavior. We used mixed effects logistic regression to determine the odds of finding at least one negative descriptor as a function of the patient’s race or ethnicity, controlling for sociodemographic and health characteristics. compared with White patients, Black patients had 2.54 times the odds of having at least one negative descriptor in the history and physical notes. Our findings raise concerns about stigmatizing language in the EHR and its potential to exacerbate racial and ethnic health care disparities.

There is robust evidence of unequal treatment by race in the US health care system and of its negative impact on patients. During 2005–13, 12.3 percent of Black respondents reported discrimination in health care compared with 2.3 percent of White respondents in a nationally representative study of the Centers for Disease Control and Prevention’s Behavioral Risk Factor Surveillance System.^1^ In 2020, four national surveys found that 11–20 percent of Black adults reported experiencing discrimination in health care during the preceding year.^2^ Although no nationally representative studies have quantified the prevalence of implicit bias (the unconscious attitudes and stereotypes that individuals may hold), multiple studies have nevertheless documented evidence of its impacts in health care. For example, Lisa Cooper and colleagues used audio recordings of health care encounters and found that physicians who tested higher on implicit bias measures were more verbally dominant and used less patient-centered language with Black patients.^3^

Studies have also identified ways in which implicit bias can negatively affect the patient-provider relationship.^4,5^ Studies using the Implicit Association Test, a tool used to measure unconscious bias, found that health care bias was associated with lower levels of patient adherence to treatment plans and lower trust in health care providers.^6,7^ In a study by Janice Blanchard and Nicole Lurie, patients who perceived racial discrimination in health care were more likely to delay care, less likely to receive recommended chronic disease screening, and less likely to follow their physician’s recommendations.^8^ Implicit bias has clear negative effects on provider communication, trust in medical care, and the delivery of health care to racially marginalized populations. Hence, it is not surprising that Black adults are more likely to report medical distrust^9^ and that medical distrust has been found to partially mediate associations between Black race and COVID-19 vaccine declination.^10^

Racial disparities in health and health care during the COVID-19 pandemic have brought additional attention to how structural racism (differential access to goods, services, or opportunities based on race) can affect patient care.Yet despite greater recognition of the potential for clinician bias in health care delivery,^11^ few studies have quantified clinician bias or examined how racism and bias are communicated among health care providers in clinical settings. Explicitly stigmatizing language such as “sickler,” “frequent-flyer,” and other terms persist in everyday medical language^12-14^ and may have consequences for patient care. In a study by Anna Goddu and colleagues, clinical vignettes were used to examine the effects of explicitly stigmatizing language on providers’ perceptions of the patient and corresponding treatment plans.^15^ The study found that when medical providers were shown a hypothetical chart note containing stigmatizing language, they were more likely to have a negative perception of the patient’s pain and to formulate a less aggressive pain management plan than when presented with a chart note without stigmatizing language. To our knowledge, no study to date has used a quantitative approach to specifically examine differences in providers’ use of negative patient descriptors by race or ethnicity in the context of real-world medical notes.

We used machine learning techniques to analyze potentially stigmatizing language in the electronic health records (EHRs) of patients seen at an urban academic medical center. Our study aimed to examine medical providers’ use of negative patient descriptors in the history and physical notes and whether use varied by patient race or ethnicity. We hypothesized that chart notes in the EHR with stigmatizing language may be disproportionately applied to racially minoritized patients. Such a pattern of disproportionate use may indicate systemic biases in a health care delivery system against racially minoritized patients. Understanding how medical providers describe and document racially minoritized patients may inform how we address racial bias in health care.

## Study Data And Methods

### DATA AND SAMPLE

We conducted a cross-sectional study of 18,459 patients with EHR data in a COVID-19 data mart at a large urban academic medical center in Chicago, Illinois. These data included health records for all patients who received medical treatment in an emergency department (ED), inpatient, or outpatient setting and who were tested for COVID-19 between January 1 and October 1, 2020. Because universal COVID-19 testing went into effect at this medical center April 30, 2020, the sample reflected all patients treated in an ED or inpatient setting between April 30 and October 1, 2020. We used the COVID-19 data mart because it contained high-quality data updated daily and because 83.3 percent of patients in our sample had at least one encounter during the five-month period with universal testing. The bulk of our sample, therefore, was not subject to the selection bias associated with symptom-based testing for COVID-19. The data mart also includes data on patients’ encounters up to one year before their first COVID-19 test, for a final study period of January 1, 2019–October 1, 2020.

Our study population included all patients with at least one history and physical note in their EHR that was entered when they were seen in an ED, inpatient, or outpatient setting. The history and physical note is written by medical providers to document the patient’s reason for seeking medical care; summarize the patient’s medical, family, and social history; and describe the plan to address the patient’s medical problems. We focus here on the history and physical note because it is intended to document a comprehensive narrative about a patient and because other providers extract relevant information from it for inclusion in their own chart notes, such as progress notes or discharge summaries. History and physical notes were extracted and deidentified before analysis. If a patient had multiple history and physical notes, all such notes were extracted and included for analysis.

We excluded patients with *International Statistical Classification of Diseases and Related Health Problems*, Tenth Revision (ICD-10), codes for dementia (*n* = 647), as negative descriptors may be applied to them more frequently because of the nature of their illness. However, we included patients with diagnoses such as substance use disorders and mental health conditions, as these diagnoses may be associated with negative unconscious bias. A list of ICD-10 codes used for each medical condition is in online appendix exhibit A1.^16^ Of the 21,001 patients who met initial inclusion criteria, we excluded 1,564 patients for missing race or ethnicity data and an additional 978 patients for missing covariate data. Our final sample consisted of 18,459 patients with 40,113 history and physical notes for analysis. The study was approved with a waiver of informed consent from the University of Chicago Institutional Review Board.

### CLASSIFICATION OF NEGATIVE DESCRIPTORS

We generated an initial list of negative patient descriptors by literature search for “difficult patient” and similar keywords. An expert panel from the Health Equity Commission of the Society of General Internal Medicine further reviewed and refined this list. Fifteen descriptors were selected for inclusion in the analysis: (non-)adherent, aggressive, agitated, angry, challenging, combative, (non-)compliant, confront, (non-)cooperative, defensive, exaggerate, hysterical, (un-)pleasant, refuse, and resist. We adjusted the descriptors to permit identification of alternative grammatical forms (for example, “adher” for “adherent,” “adhere,” or “adhered).

We preprocessed history and physical notes using natural language processing techniques to standardize the text data and split notes into sentences.^17^ From all sentences in the data set, we selected a random sample of sentences containing one or more of the fifteen selected patient descriptors for manual review and annotation by the lead author under the direction of two clinician researchers and a natural language processing methodologist. We categorized the use of each descriptor in one of three possible ways: negative (for example, “[the patient] has been poorly compliant” or “uncooperative with his physical exam” or “is non-adherent with her medication”), positive (for example, “[the patient] has been compliant” or “is calm and cooperative with interview” or “reports adherence with home medications”), or out of context (for example, “using a non-compliant balloon” or “airway semi-cooperative” or “non-adherent bandage”). Use in a sentence was considered out of context if the descriptor was applied to something other than the patient or a specific interaction with the patient. The list of patient descriptors and examples of use in different contexts are in appendix exhibit A2.^16^ A total of 6,818 sentences were classified and used to inform the machine learning model.

### DEVELOPMENT OF THE MODEL

We used natural language processing and machine learning methods to develop the model to analyze the clinical notes data set. The goal of this model was to analyze a sentence containing a patient descriptor and determine the context of the descriptor (negative, positive, or out of context). We divided the manually labeled sentences as follows: two-thirds into a training set to train the model and the remaining one-third into a testing set for evaluation purposes. The trained model interpreted the sentences from the testing set and predicted their context as negative, positive, or out of context. Based on the testing set, the model correctly predicted the context of a sentence with a macro average value F1 of 0.935 (a perfect F1 score is 1).^18^ We then applied the final model to all chart notes in the data set. Additional information on the model development and sample code are in appendix exhibits A3 and A4.^16^

### STUDY VARIABLES

For our primary analysis, the dependent variable was the occurrence of at least one negative descriptor in a patient’s history and physical note. The independent variable was each patient’s race and ethnicity as recorded in the EHR. For this analysis, we designated “White” to be non-Hispanic White, “Black” to be non-Hispanic Black or African American, “Hispanic or Latino” as any patient identifying as Hispanic or Latino, and “other” to be patients of any other racial or ethnic identities. Race and ethnicity data are typically queried and recorded in the EHR by a registration clerk before the patient encounter.

Our study’s covariates included patient age (0–17, 18–29, 30–44, 45–64, or 65+), sex (male or female), insurance provider (Medicaid, Medicare, or employer-based/private), marital status (married or unmarried), primary language (English or not English), COVID-19 test result (positive or negative), encounter location (inpatient, outpatient, or ED), non-age-adjusted Charlson Comorbidity Index, encounter length (days), and timing of encounter either before March 1, 2020 (before the COVID-19 pandemic began), or after March 1, 2020 (after the COVID-19 pandemic began).

We adjusted for sociodemographic characteristics that have known associations with patient care as well as medical complexity, based on prior literature indicating that patients with these attributes may be perceived during clinical encounters as more difficult.^19^ We adjusted for timing of encounter because our study period included data from before and after the start of the COVID-19 pandemic (designated as March 1, 2020), providing us with an opportunity to also examine negative descriptor use specifically during the pandemic. We adjusted for encounter location in case the setting (inpatient, outpatient, or ED) significantly affected the use of a negative descriptor.

### STATISTICAL ANALYSIS

For our primary analysis, we fit multilevel mixed-effects logistic regression models to determine the odds of a negative patient descriptor in each note as a function of race or ethnicity (using non-Hispanic White as the referent group). Multilevel modeling enabled analysis at the note level, with notes nested within encounters and encounters nested within patients (that is, a random effect for both encounter and patient). It also enabled adjustment for covariates at all three levels. We provided unadjusted estimates out of concern that adjusting for variables affected by structural inequalities in health and health care (for example, insurance type and comorbidities) may inappropriately minimize our estimation of disparities. Data analysis was conducted using STATA, version 16.1.

### LIMITATIONS

Our study had several limitations. First, it was performed at a single urban academic medical center, limiting generalizability. The machine learning model would be ideally validated on patient notes from multiple institutions across the US.

Second, a small proportion (16.7 percent) of the sample may have been prone to selection bias as the sample comprised patients who were tested for COVID-19 before the implementation of universal testing. This group may have been more likely to have a usual source of care and access to testing, although community-based outreach likely limited this effect. Ultimately, the majority of patients (83.3 percent) were included after universal testing was implemented and reflect all patients treated at the medical center on or after April 30, 2020.

Third, limited racial and ethnic heterogeneity in the sample prevented further disaggregation by either race or ethnicity to include additional groups in our analysis (for example, Asian race).

Fourth, this study was conducted in the years immediately preceding and following the onset of the COVID-19 pandemic, further limiting generalizability. Especially during the first wave of the pandemic, clinicians were functioning under exceptional circumstances, which likely altered the way they communicated and interacted with patients.We thus include analyses examining the timing of encounter relative to the onset of the pandemic.

Fifth, the natural language processing and machine learning algorithm may have missed or falsely detected a small percentage of negative descriptors, although the macro average value F1 metric was high (0.935 out of a perfect score of 1).

Sixth, despite literature documenting the use of words such as “defensive,” “hysterical,” and “unpleasant,” we did not observe the use of these descriptors at a significant frequency in the sample population. The machine learning results may also be partly influenced by trends in the training data, limiting identification of infrequently used descriptors.

Last, we recognize that the use of negative descriptors might not necessarily reflect bias among individual providers; rather, it may reflect a broader systemic acceptability of using negative patient descriptors as a surrogate for identifying structural barriers. Use of the term “noncompliant,” for instance, does not carry neutral connotations, but race-based differences in treatment compliance often reflect underlying structural challenges (for example, medical distrust or financial hardship) rather than individual patient motivations or behaviors. The application of such terms thus can stigmatize patients for factors outside of their control, regardless of the ontology of bias.

## Study Results

### DESCRIPTIVE STATISTICS

Our sample consisted of 18,459 patients (exhibit 1), 33,142 unique encounters (exhibit 2), and 40,113 history and physical chart notes (data not shown). Almost one-third (29.7 percent) of the patients were White, 60.6 percent were Black, 6.2 percent were Hispanic or Latino, and 3.5 percent were categorized as other. The mean age was 47.4 years (SD 23.0; data not shown), and 56.0 percent were female (exhibit 1). In total, 8.2 percent of patients had one or more negative descriptors recorded in the history and physical notes in their EHR (data not shown). Exhibits 1 and 2 display the full descriptive statistics of the study population and encounter characteristics.

### NEGATIVE DESCRIPTORS AND RACE/ETHNICITY

The most commonly used descriptors in any contexts were “refused” (*n* = 1,461), “(not) adherent” (*n* = 605), “(not) compliant” (*n* = 561), and “agitated” (*n* = 409) (data not shown). In adjusted models, Black patients had 2.54 times the adjusted odds (95% confidence interval: 1.99, 3.24) of having one or more negative descriptors in the EHR compared with White patients (exhibit 3). In addition, patients with Medicaid (adjusted odds ratio: 2.66; 95% CI: 2.08, 3.40) or Medicare (AOR: 2.08; 95% CI: 1.57, 2.75) insurance had higher adjusted odds of a negative descriptor compared with patients with private or employer-based insurance. Unmarried patients had higher adjusted odds of a negative descriptor (AOR: 2.12; 95% CI: 1.70, 2.65) compared with married patients. Increased Charlson Comorbidity Index (AOR: 1.11; 95% CI: 1.07, 1.15) was also associated with higher adjusted odds of a negative descriptor. In contrast, notes written after March 1, 2020 (AOR: 0.82; 95% CI: 0.70, 0.96), and in the outpatient setting (AOR = 0.37; 95% CI: 0.31, 0.45) had lower odds of having a negative descriptor. There were no statistically significant interactions between covariates (data not shown).

In addition, we performed a sensitivity analysis excluding patients with ICD-10 codes related to delirium, substance use, or other mental and behavioral diagnoses, as these patients may be more likely to have negative descriptors applied for condition-related reasons. Results were substantively similar, with Black patients having 2.88 times the adjusted odds (95% CI: 2.03, 4.11) of having a negative descriptor compared with White patients (appendix exhibit A5).^16^

In patient-level sensitivity analyses using the number of negative notes per patient (appendix exhibits A6 and A7),^16^ Black race was associated with 5.6 additional negative notes per 100 patients (95% CI: 3.5, 7.8) relative to White race.

## Discussion

In this study conducted at an urban academic medical center, we found that Black patients had 2.54 times the odds of being described with one or more negative descriptors in the history and physical notes of their EHRs, even after we adjusted for their sociodemographic and health characteristics. Our findings suggest disproportionate use of negative patient descriptors for Black patients compared with their White counterparts, which raises concerns about racial bias and possible transmission of stigma in the medical record.

Research and editorial writings by medical providers attest to the common use of terms such as “difficult,” “challenging,” and “resistant” to describe patients.^20-22^ These and similar descriptors are not explicitly stigmatizing terms, but they may impart a negative connotation in the context of describing a patient. Jenny Park and colleagues used qualitative methods to analyze medical charts and documented five common types of negative language, which included portraying patients as difficult and stereotyping on the basis of race or social class.^23^ Goddu and colleagues observed in their study of hypothetical chart notes that explicitly stigmatizing language (that is, language that conjured up negative stereotypes) negatively affected respondents’ attitudes toward the patient and resulted in less aggressive pain management plans.^15^

Our findings are especially alarming because we limited our evaluation of negative descriptors to the history and physical notes of patient EHRs. In a study by Michael Wang and colleagues, only 18 percent of text in inpatient progress notes were originally manually input, with the majority being imported from prior documentation.^24^ History and physical notes provide key information frequently drawn on by other care providers. Negative descriptors written in the admission history and physical may be likely to be copied into subsequent notes, recommunicating and amplifying potential biases. This practice underscores the responsibility of providers who document the initial patient encounter to do so in an aware and sensitive manner.

Of interest, our results suggest that outpatient encounters were associated with lower adjusted odds of having a negative descriptor in the EHR, which may indicate protective factors that are more prevalent in the outpatient clinical setting than in the inpatient setting. For example, previous research has found that physicians may be at increased risk of using stereotypes as a cognitive shortcut in stressful clinical environments characterized by time pressure, increased cognitive burden, and decreased resources.^25^ Outpatient care may also be less prone to negative descriptor use because encounters involve one-to-one patient-provider communication in ongoing, often long-term relationships.

Contrary to expectations, notes written after the COVID-19 pandemic began were associated with decreased odds of having a negative descriptor in the EHR. The onset of the pandemic coincided with a historically defining moment of national response to racialized state violence (for example, the police murders of George Floyd and others) and revealed stark racial disparities in COVID-19 health access and outcomes. These social pressures may have sensitized providers to racism and increased empathy for the experiences of racially minoritized communities. Although such a shift may have contributed to reductions in negative descriptor use after March 1, 2020, additional research is required to understand which aspects of the COVID-19 pandemic affected physicians’ language. For instance, it may be that health care providers had less frequent interactions with patients, reducing opportunities for conflict to develop. Alternatively, patients being treated for COVID-19 may have been considered “less at blame” for their illness compared with patients with other more chronic and lifestyle-associated conditions.

Future research is needed to investigate the longitudinal consequences of a negative descriptor in a patient’s medical record. Our study demonstrates the disproportionate application of negative descriptors to the history and physical notes of Black patients, but it cannot characterize relationships between an initial negative descriptor and future occurrences of negative descriptors. Our study also does not characterize potential impacts on a patient’s medical care.

We theorize that negative descriptors in a patient’s EHR may assign negative intrinsic value to patients. Subsequent providers may read, be affected by, and perpetuate the negative descriptors, reinforcing stigma to other health care teams. It is also plausible that if a provider with implicit biases were to document a patient encounter with stigmatizing language, the note may influence the perceptions and decisions of other members of the care team, irrespective of the other team members’ biases or lack thereof. Additional investigation may use a similar machine learning approach to examine EHR data over a longer period of time for repeated use of negative descriptors and for potential effects on health outcomes. Similar to the current study, this approach would also be limited to investigation of documented data and would not be able to assess nondocumented bias (for example, oral presentations) or outcomes such as patient trust.

## Policy Implications

Our findings suggest multiple opportunities for policy interventions to address the use of negative descriptors. First, medical institutions can better address the introduction of implicit bias of all forms, but especially racial bias. Negative descriptors enter the chart either by a note writer who introduces negative language or by someone who perpetuates previously used language. For example, a provider’s use of the term “aggressive” to describe a Black male patient may reflect the provider’s own personal bias about Black men. But once this stigmatizing label becomes attached to a patient in the medical record, it potentially affects the perceptions and decisions of future providers regardless of whether future providers hold a preexisting bias about Black men being aggressive.

The goal of addressing implicit bias is to address the underlying mechanisms that prompt the use of negative descriptors to describe patients. This includes preventing the introduction of biased language by providers, preventing the perpetuation of biased language by members of the health care team, and increasing awareness of the effects of providers’ language on the patient relationship. Interventions may include provider bias training and addressing health care system factors that may predispose providers toward expressions of bias.

Provider bias training can include competencies in nonstigmatizing language for interprofessional communication. Use of “people-first” language (for example, saying a patient has an “alcohol use disorder” instead of labeling them an “alcoholic”) is becoming more common,^12,14^ but such changes in vocabulary do not address the potential for contextual stigmatization. Better education on race and racism may help equip providers with the understanding needed to identify, prevent introduction of, and discontinue use of negative descriptors in the EHR. Nevertheless, as more institutions begin to share electronic records, a broader shift may be necessary to catalyze evolution in the language of health care. Regulatory bodies, such as the Accreditation Council for Graduate Medical Education and its counterparts, maintain training standards regarding professional communication, internal biases, and nondiscrimination.^26^ Although these guidelines describe and affirm the importance of nonstigmatizing, patient-centered language, specific recommendations may be necessary to align professional standards with practices and prevent the transmission of bias across institutions.

The need to review professional language standards in medicine is all the more pressing given implementation of OpenNotes policies, which allow patients full access to their EHRs, including chart notes. In a mixed-methods analysis of oncologists’ notes, Jordan Alpert and colleagues found that note text did not significantly vary between pre- and postimplementation of OpenNotes software.^27^ In a study by Leonor Fernández and colleagues, patients were shown notes from the OpenNotes EHR, and 10.5 percent reported feeling judged or offended by the notes’ contents.^28^ Despite patient observation, providers may be unable to change their language without self-awareness and training on potential biases. The ongoing implementation of OpenNotes should encourage both providers and institutions to seriously consider the language used to describe patients or else risk harming the patient-provider relationship with downstream effects on patient satisfaction, trust, and even potential litigation.

In addition, hospital medicine can identify and address structural factors of health care delivery that exacerbate the use of stereotypes. In a study by Liselotte Dyrbye and colleagues, symptoms of burnout were associated with greater explicit and implicit biases among resident physicians.^29^ Addressing contributors to burnout is a necessary intervention. Emphasizing providers’ responsibility to change without addressing health care system issues could increase burnout and inadvertently exacerbate bias. Alternatively, delivery models that increase inpatient continuity of care may replicate some protective factors associated with outpatient encounters.^30^

## Conclusion

We found that Black patients at an urban academic medical center had disproportionately higher odds of negative patient descriptors appearing in the history and physical notes of their EHRs compared with White patients. This difference may indicate implicit racial bias not only among individual providers but also among the broader beliefs and attitudes maintained by the health care system. Such bias has the potential to stigmatize Black patients and possibly compromise their care, raising concerns about systemic racism in health care.

## Supplementary Material

Supplemental Appendix

## Figures and Tables

**EXHIBIT 1:** Use of negative patient descriptors in electronic health records (EHRs), by characteristics of patients from a large urban academic medical center in Chicago, Illinois, January 2019-October 2020

			Negative patient descriptors in EHRs			
	Patients (*N* = 18,459)		None (*n* = 16,938)		One or more (*n* = 1,521)	
Patient characteristics	%	No.	%	No.	%	No.
Age, years						
0–17	11.9	2,197	12.0	2,028	10.7	169
18–29	13.4	2,464	13.5	2,276	11.9	188
30–44	16.8	3,101	17.0	2,862	15.1	239
45–64	30.3	5,591	30.0	5,057	33.7	534
65+	27.7	5,106	27.6	4,649	28.8	457
Sex						
Female	56.0	10,327	56.4	9,514	51.2	813
Race and ethnicity						
Non-Hispanic White	29.7	5,479	31.2	5,263	13.6	216
Non-Hispanic Black	60.6	11,192	58.8	9,928	79.7	1,264
Hispanic or Latino	6.2	1,152	6.3	1,070	5.2	82
Other	3.5	636	3.6	611	1.5	25
Marital status						
Not married	67.8	12,517	66.5	11,224	81.5	1,293
Primary language						
Not English	2.2	399	2.2	369	1.9	30
Insurance						
Medicaid	32.2	5,950	31.0	5,232	45.2	718
Medicare	32.7	6,026	32.0	5,404	39.2	622
Employer-based/private	35.1	6,483	37.0	6,236	15.6	247
COVID-19 test result						
Positive	8.2	1,521	8.1	1,363	10.0	158

**source** Authors’ analysis of data from the University of Chicago Center for Research Informatics COVID-19 data mart. **note** Results reflect detection of a negative patient descriptor (none versus at least one) in the patient’s history and physical notes.

**EXHIBIT 2:** Use of negative patient descriptors in electronic health records (EHRs) of a large urban academic medical center in Chicago, Illinois, by encounter characteristics, January 2019-October 2020

			Negative patient descriptors in EHR			
	Encounters (*N* = 33,142)		None (*n* = 30,289)		One or more (*n* = 2,853)	
Encounter characteristics	% or mean	No. or SD	% or mean	No. or SD	% or mean	No. or SD
Charlson Comorbidity Index (mean and SD)	1.4	0.01	1.6	0.01	2.4	0.03
Encounter length, days (mean and SD)	4.4	0.04	5.8	0.06	7.5	0.1
Timing of encounter^a^ (% and number)						
Before March 1, 2020	19.3	6,401	17.1	5,718	26.0	1,740
On or after March 1, 2020	80.7	26,741	82.9	27,690	74.1	4,965
Encounter location (% and number)						
Inpatient	46.6	15,459	50.7	16,928	68.0	4,556
Outpatient	51.8	17,157	48.0	16,018	31.0	2,075
Emergency department	1.6	526	1.4	462	1.1	74

**source** Authors’ analysis of data from the University of Chicago Center for Research Informatics COVID-19 data mart. **notes** Results reflect detection of a negative patient descriptor (none versus at least one) in the patient’s history and physical notes. SD is standard deviation.

a We selected March 1, 2020 as an approximate date at which providers’ behavior may have changed in conjunction with the COVID-19 pandemic. The first cases in Illinois that were not linked to travel from China were reported February 29 and March 2, 2020; see Illinois Department of Public Health [Internet]. Springfield (IL): IDPH. Press release, State of Illinois public health officials announce new presumptive positive COVID-19 case in Illinois; 2020 Feb 29 [cited 2021 Dec 8]. Available from: https://dph.illinois.gov/resource-center/news/2020/march/state-illinois-public-health-officials-announce-new-presumptive-positive-covid-19-caseillinois.html. Local medical institutions began implementing COVID-19 measures, with government measures to follow shortly thereafter.

**EXHIBIT 3:** Association of negative patient descriptor use in electronic health records with patient and encounter characteristics at a large urban academic medical center in Chicago, Illinois, odds ratios, January 2019-October 2020

Characteristics	Unadjusted odds ratio	Adjusted odds ratio
Race and ethnicity (ref: non-Hispanic White)		
Non-Hispanic Black	2.84****	2.54****
Hispanic or Latino	1.34***	1.51*
Other	0.89	1.07
Age, years (ref: 65+)		
0–17	0.99	0.81
18–29	1.23***	0.87
30–44	1.19**	1.12
45–64	1.23****	1.40****
Sex (ref: male)		
Female	0.93*	0.76****
Marital status (ref: married)		
Not married	2.30****	2.12****
Language (ref: English)		
Not English	0.61***	0.77
Insurance provider (ref: employer-based/private)		
Medicaid	3.00****	2.66****
Medicare	2.15****	2.08****
Encounter location (ref: inpatient)		
Outpatient	0.26****	0.37****
Emergency department	0.85	0.70
Charlson Comorbidity Index	1.08****	1.11****
Encounter length (days)	1.01*	1.00
Positive COVID-19 test	1.16*	0.88
Timing of encounter (ref: before March 1, 2020)^a^		
On or after March 1, 2020	0.77****	0.82**

**source** Authors’ analysis of data from the University of Chicago Center for Research Informatics COVID-19 data mart. **notes** Results reflect detection of a negative patient descriptor (none versus at least one) in the patient’s history and physical notes. Reference value is 1.

a We selected March 1, 2020 as an approximate date at which providers’ behavior may have changed in conjunction with the COVID-19 pandemic. See note a in exhibit 2 for details.

* *p* < 0.10

** *p* < 0.05

*** *p* < 0.01

**** *p* < 0.001
