# Supplementary material for: Negative Patient Descriptors: Documenting Racial Bias In The Electronic Health Record
Source: Health Aff (Millwood). Author manuscript; Available in PMC 2023 Feb 1. (PMC8973827; doi:10.1377/hlthaff.2021.01423)
Supplement: Supplemental Appendix [file NIHMS1789065-supplement-Supplemental_Appendix.pdf]

APPENDIX

Appendix Exhibit A1. Medical Conditions and Corresponding ICD10 Codes

Diagnosis or Diagnosis Category	ICD10 Codes
AIDS	B20; B21; B22; B24
Cancer	C00-26; C30-34; C37-41; C43; C45-58; C60-85; C88, C90-99
Cerebrovascular disease	G45; G46; H34; I60-69
Chronic kidney disease	I12.0; I13.1; N03; N05; N18; N19; N25.0; Z94; Z99.2
Chronic obstructive pulmonary disease	I27; J40-44; J46; J47; J60-68; J70
Congestive heart failure	I09; I10; I11; I13; I25; I42; I43; I50; P29
Connective tissue disease	M05; M06; M31; M32; M33; M34; M35; M36.0
Delirium	R41
Dementia	F01; F02; F03; G30; G31
Diabetes mellitus	E10-14
Hemiplegia	G04.1; G11.4; G80; G81; G82; G83
Liver disease	B18; I85; I86.4; I98; K70; K71; K72; K73; K74; K76
Mental, behavioral, and neurodevelopmental disorders	F01-99
Myocardial infarction	I21; I22; I25
Peptic ulcer disease	K25; K26; K27; K28
Peripheral vascular disease	I70; I71; I73; 77; I79; K55; Z95

Source: International Classification of Diseases, Tenth Revision, Clinical Modification (ICD-10-CM). 10th ed. Geneva: World Health Organization; 2021.

Appendix Exhibit A2. Negative Descriptor Use in Medical Records and Example Sentences

	Negative sentences	Positive / Out-of-context sentences
**All descriptors**		
**(non) Adherent**	"Her entire course has been difficult, non-adherence to clinic visits and prescribed treatment."	"Adherent to mouthwash regimen and skin care regimen."
**Aggressive**	"...became extremely aggressive upon inquiring about polysubstance abuse."	"Will continue to provide aggressive asthma therapies..."
**Agitated**	"Behavior is agitated and aggressive."	"Non agitated on ventilator."
**Angry**	"Awake, alert, angry at having pain medication interrupted this morning while somnolent."	Example not available.
**Challenging**	"The patient has an extremely long and complicated history of frequent, challenging admissions and opiate use disorder."	"Technically very challenging study due to patient body habitus."
**Combative**	"On arrival to the trauma bay, the patient was combative and aggressive..."	"She is not agitated, not aggressive and not combative."
**(non) Compliant**	"…has been poorly compliant with follow up and recommendations was lost to follow-up…"	"Prior to these symptoms over last few days the patient was at baseline and has been compliant with his medications"
**Confront**	"He frequently leaves against medical advice and reports this is due to getting into confrontations with staff"	" Visual fields are full to confrontation."
**(non) Cooperative**	"She is uncooperative."	"She is calm and cooperative with interview."
**Defensive**	Example not available.	Example not available.
**Exaggerate**	"Exaggerated pain out of proportion to exam"	"No exaggerated curvature of the lumbar spine."
**Hysterical**	Example not available.	Example not available.
**(not) Pleasant**	Example not available.	"Awakens easily to exam, pleasant, cooperative."
**Refuse**	"…patient seen by sleep medicine and decision back then was to place patient on bipap but she refused"	Example not available.
**Resist**	"distressed anxious resistant to exam	"Prior cultures reviewed no resistant infections."

Source: Authors' analysis of data from the University of Chicago Center for Research Informatics COVID-19 Datamart.

Appendix Exhibit A3. Detailed Methods on the Development of the Machine Learning Model

NLP and machine learning methods were used to develop the model to analyze the clinical notes dataset. The notes were sentence split with NLTK.(1) Each sentence was pre-processed with standard natural language processing techniques, the main ones were: tokenization, lowercasing, replacement of numbers with the token "number", and removal of tokens without any alphanumeric content.

The goal of the machine learning model was to analyze a sentence containing a patient descriptor and determine the context of the descriptor (negative, positive, or out-of-context). Sentences without a descriptor were considered "out-of-context" by the model. Using a scikit-learn pipeline, the sample of classified sentences were divided into n-grams with a size of 1 (individual words) to 3 (adjacent words) and transformed into a bag-of-words vector representation.(2) This process derives a pattern of word order and frequency for a given sentence with a descriptor, creating a sparse numerical count vector of the same length for each sentence. These vectors can then be associated with their sentence's context. Since some words appear more frequently than others (e.g. articles, conjunctions), vector representations were weighted with the term frequency-inverse document frequency approach to emphasize relevant words and phrases according to this heuristic.(3) A sample of the pipeline code can be viewed in Appendix Exhibit A3.

Data from the ground-truth labeled sentences was divided into a training set (2/3) and testing set (1/3). Data from the training set was used to create a linear model with stochastic gradient descent learning and an elastic net regularization.(4) This model interpreted the vector

representation of a sentence and predicted its context as negative, positive, or out-of-context. In an iterative manner, the number of sentences was increased, and incorrect predictions were manually corrected to arrive at the final labeled dataset described above. On the training set, hyperparameter optimization and 10-fold cross-validation was performed to find the best alpha parameter for the model. This model was evaluated on the testing set and correctly predicted the context of a sentence with a macro average value F1 of 0.935 (a perfect F1 score is 1).(5) Finally, the model was trained on all ground-truth labeled notes with the best alpha parameter found, and then applied to all chart notes in the dataset.

Citations

1. Bird S, Klein E, Loper E. Natural Language Processing with Python. 1st ed. O'Reilly Media, Inc; 2009.

2. Pedregosa F, Varoquaux G, Gramfort A, Michel V, Thirion B, Grisel O, et al. Scikit-learn: Machine Learning in Python. MACHINE LEARNING IN PYTHON. :6.

3. Bollig N, Clarke L, Elsmo E, Craven M. Machine learning for syndromic surveillance using veterinary necropsy reports. PLOS ONE. 2020 Feb 5;15(2):e0228105.

4. Marafino BJ, John Boscardin W, Adams Dudley R. Efficient and sparse feature selection for biomedical text classification via the elastic net: Application to ICU risk stratification from nursing notes. Journal of Biomedical Informatics. 2015 Apr 1;54:114–20.

5. Savova GK, Masanz JJ, Ogren PV, Zheng J, Sohn S, Kipper-Schuler KC, et al. Mayo clinical Text Analysis and Knowledge Extraction System (cTAKES): architecture, component evaluation and applications. J Am Med Inform Assoc. 2010;17(5):507–13.

Appendix Exhibit A4. Sample Code Utilized for TF-IDF Vectorizer

```
from sklearn.feature_extraction.text import TfidfVectorizer
from sklearn.pipeline import Pipeline
from sklearn.model_selection import GridSearchCV
from sklearn.linear_model import SGDClassifier

wordvect = TfidfVectorizer(analyzer='word')
classifier = SGDClassifier(loss='log',
                           class_weight='balanced',
                           penalty='elasticnet',
                           random_state=SEED,
                           max_iter=1000,
                           tol=0.001)

pipeline = Pipeline([('vect', wordvect), ('classifier',
classifier)])

scoring = 'f1_weighted'
parameters = {'vect__stop_words' : [None],
              'vect__ngram_range': [(1, 3)],
              'vect__lowercase': [True],
              'vect__min_df': [2],
              'vect__max_df': [0.8],
              'vect__sublinear_tf': [True],
              'classifier__alpha' : [0.000001, 0.000005,
0.00001, 0.00005, 0.0001, 0.0005, 0.001, 0.005, 0.01],
              'classifier__l1_ratio' : [0.85]
             }
```

Source: Code developed by author Tomasz Oliwa, Ph.D.

Appendix Exhibit A5. Patient Descriptors and Patient Race/Ethnicity, Sensitivity Analysis Excluding Mental and Behavioral Diagnoses

Variable	Negative Descriptor	
	Unadjusted OR	Adjusted OR
Race/Ethnicity (ref: white non-HL)		
Black, non-HL	2.47 ****	2.88 ****
Hispanic/Latino	1.17	1.39
Other	0.88	1.30
Age, years (ref: 65+)		
0-17		1.03
18-29		1.03
30-44		1.13
45-64		1.05
Sex (ref: Male)		
Female		0.89
Marital status (ref: married)		
Not married		2.17 ****
Language (ref: English)		
Not English		1.05
Insurance provider (ref: private/employer-based)		
Medicaid		2.24 ****
Medicare		1.88 ****
Encounter Location (ref: Inpatient)		
Outpatient		0.33 ****
Emergency		0.42 **
Charlson Comorbidity Index		1.17 ****
Encounter length (days)		1.00
COVID-19 (+)		0.80
Timing of encounter (ref: pre 3/1/2020)		
After 3/1/2020		0.76 **

Source: Authors' analysis of data from the University of Chicago Center for Research Informatics COVID-19 Datamart.
*p<0.10, **p<0.05, ***p<0.01, ****p<0.001

Appendix Exhibit A6. Mean Negative Notes and Sentences by Race/Ethnicity

Note Characteristics	All	White, non-Hispanic/Latino	Black, non-Hispanic/Latino	Hispanic/Latino	Other
Number of Notes	2.17	2.14	2.15	2.62	2.11
Negative Notes	0.049	0.021	0.066	0.037	0.027
Negative Sentences	0.128	0.061	0.168	0.097	0.052

Source: Authors' analysis of data from the University of Chicago Center for Research Informatics COVID-19 Datamart.

Appendix Exhibit A7. Negative Notes and Patient Race/Ethnicity, Patient-Level Sensitivity Analysis

Variable	Negative Descriptor
	Adjusted OR
Race/Ethnicity<br>(ref: white non-HL)	
Black, non-HL	0.056 ****
Hispanic/Latino	-0.019
Other	-0.0063
Age, years (ref: 65+)	
0-17	-0.030
18-29	0.0026
30-44	0.031 *
45-64	0.031 **
Sex (ref: Male)	
Female	-0.0088
Marital status<br>(ref: married)	
Not married	0.048 ****
Language (ref: English)	
Not English	-0.038
Insurance provider<br>(ref: private/employer-based)	
Medicaid	0.064 ****
Medicare	0.033 **
Encounter Location<br>(ref: Inpatient)	
Outpatient	-0.039 ****
Emergency	-0.0073
Charlson Comorbidity Index	0.0058 **
Total number of notes	0.081 ****

Source: Authors' analysis of data from the University of Chicago Center for Research Informatics COVID-19 Datamart.
*p<0.10, **p<0.05, ***p<0.01, ****p<0.001
